# Comparison of the Mechanical Properties and Surface Characteristics of Vat Photopolymerization Resin Materials and a Polymethyl Methacrylate Disc Material

**DOI:** 10.3390/ma19112220

**Published:** 2026-05-25

**Authors:** Fei Yu, Ryuhei Kanda, Yoshiya Hashimoto, Kazuhiko Suese, Koji Mitamura, Yasuyuki Kobayashi, Kosuke Kashiwagi

**Affiliations:** 1 Department of Fixed Prosthodontics and Occlusion, Osaka Dental University, 8-1 Kuzuhahanazonocho, Hirakata 573-1121, Osaka, Japan; yf609576804@163.com (F.Y.); fpd-kk@cc.osaka-dent.ac.jp (K.K.); 2 Division of Creative and Integrated Medicine, Advanced Medicine Research Center, Translational Research Institute for Medical Innovation (TRIMI), Osaka Dental University, 8-1 Kuzuhahanazonocho, Hirakata 573-1121, Osaka, Japan; 3 Department of Biomaterial, Osaka Dental University, 8-1 Kuzuhahanazonocho, Hirakata 573-1121, Osaka, Japan; yoshiya@cc.osaka-dent.ac.jp; 4 Osaka Dental University, 8-1 Kuzuhahanazonocho, Hirakata 573-1121, Osaka, Japan; 5 Osaka Research Institute of Industrial Science and Technology, Morinomiya Center, 6-50, Morinomiya-1, Joto-ku, Osaka 536-8553, Osaka, Japan

**Keywords:** vat photopolymerization, polymethyl methacrylate, mechanical properties, surface characteristics, build angle

## Abstract

Additive manufacturing using vat photopolymerization (VPP) resin materials has gained attention for fabricating dental prostheses; however, the effects of material type and build angle on their properties remain unclear. We compared the mechanical properties of two filler-containing VPP hybrid resins, SprintRay Ceramic Crown (CC) and OnX Tough 2 (OT), with those of a conventional polymethyl methacrylate (PMMA) disc material, and evaluated the influence of build angle on surface characteristics, dimensional accuracy, and mechanical performance. Specimens were fabricated using a DLP system at build angles of 0°, 45°, and 90°. Vickers hardness, surface morphology and roughness, dimensional deviations, flexural strength, elastic modulus, and fracture energy were assessed according to relevant standards. CC exhibited significantly higher hardness and elastic modulus than PMMA and OT, whereas OT showed the highest fracture energy. Surface morphology and roughness were strongly affected by build angle, with 45° producing distinct periodic patterns and increased roughness. Dimensional evaluation revealed a tendency toward overbuilding, particularly in the vertical direction at 45°. Flexural properties were also influenced by build angle, with 45° generally providing favorable performance. Both material composition and build angle affect VPP-fabricated dental resin performance, highlighting the importance of appropriate material and processing selection for clinical applications.

## 1. Introduction

In recent years, the fabrication of dental prostheses using three-dimensional (3D) printing methods developed based on additive manufacturing (AM) technology has received considerable attention in the field of dentistry [1,2,3], and applications for various purposes, such as the production of models [4,5], surgical guide plates [6,7,8], removable dentures [9,10], and fixed dental prostheses [11,12], are currently being explored. Furthermore, compared to subtractive methods, these advances involving AM technology contribute to reducing labor and time costs [13], minimizing material waste, and enabling the reproduction of more complex shapes [2,3].

Among these, fabricating techniques using resin materials manufactured via vat photopolymerization (VPP) represent a key strategic option for next-generation crown restoration procedures [14,15]; in clinical cases to date, they have primarily been applied to temporary restorations [16,17,18]. Additionally, some VPP resins have been developed using added fillers to improve their mechanical properties, and they are expected to be used for fabricating definitive restorations [12]. However, information regarding the prognosis of and indications for crown restorations made using these filler-modified VPP hybrid resin materials remain insufficient; consequently, they are currently primarily being used as temporary restorative materials [19].

Under these circumstances, two types of filler-reinforced VPP hybrid resin materials—SprintRay Ceramic Crown (CC) and SprintRay OnX Tough2 (OT)—have been approved as managed medical devices in Japan (with approval numbers 307ADBZI00038000 and 307ADBZI00037000 for CC and OT, respectively), and their clinical use is expected to increase in the future. However, there are few reports on the basic data related to these materials, and the differences in mechanical properties compared to those of existing provisional restoration materials used for milling remain unclear.

Furthermore, reports suggest that when using VPP-fabricated materials, the printing angle affects dimensional accuracy [20,21] and mechanical properties [22,23]. Other factors that may affect dimensional accuracy and mechanical strength include manufacturing methods [24], material composition [25], filler content [26], and the type of post-processing [27,28]. There is a lack of consistency in these various factors across dental 3D printing systems that use different VPP resin materials, and they may interact with the build angle in complex ways; consequently, no consensus regarding these factors has been reached among the various reports. Furthermore, while the build angle may influence surface characteristics and roughness, these surface features may contribute to plaque retention [29].

Therefore, the aims of this study were as follows: (1) to compare the mechanical properties of CC and OT with those of a conventional polymethyl methacrylate (PMMA) disc material used for milling intended for provisional restorations; and (2) to compare the surface characteristics, dimensional accuracy (length, thickness, width), and mechanical parameters in a three-point bending test of CC and OT specimens fabricated at various build angles. The null hypothesis of this study was that there would be no differences in mechanical properties among the materials, and that the build angle of VPP resin would not affect surface characteristics, dimensional accuracy, or mechanical parameters.

## 2. Materials and Methods

### 2.1. Sample Preparation

The materials used are shown in Table 1. Two types of VPP hybrid resins (Sprintray Ceramic Crown and SprintRay OnX Tough 2, both from Sprintray, Los Angeles, CA, USA) were used as VPP-fabricated materials. Additionally, a PMMA disc (Resin Discs, Yamahachi, Aichi, Japan) was used as the control material.

A low-speed diamond blade (Isomet LS, Buehler, Lake Bluff, IL, USA) was used to section the PMMA disc. The disc was cut to the specified dimensions for each experiment under running water at a rotation speed of ≤300 rpm, and the specimens obtained through these steps were designated as the PM samples. The VPP samples were prepared in accordance with the manufacturer’s instructions and our previous report [30]. Two types of VPP resin test specimens were designed using CAD software (Rhino 7 Ver 7.38.24338.17001; Robert McNeel & Associates, Seattle, WA, USA) with dimensions appropriate for each experiment. The design data were imported into the included 3D printing software (RayWare, Sprintray) in the Standard Tessellation Language (STL) format. Using a compatible VPP device (Sprintray PRO2, Sprintray, CA, USA) and programs tailored to each resin material, samples were fabricated using a layer thickness of 100 µm, dimensions appropriate for each experimental objective, and the required build angle. The support structures were removed from the fabricated objects, and they were washed using an alcohol-based cleansing solution (3D Medical Clean, Sankyo, Tokyo, Japan) for approximately 30 s; this process was repeated three times. After drying, post-processing was performed using a dedicated post-curing unit (Nano Cure, SprintRay) according to the manufacturer’s recommended protocol for each material. The resulting samples were designated as the CC and OT samples.

### 2.2. Vickers Hardness

All samples were fabricated using dimensions of 10 mm on each side and a thickness of 3 mm. The samples were prepared in accordance with the manufacturer’s instructions and our previous report [30]. The build angles for both CC and OT were set to 0 degrees relative to the platform. Five samples were prepared for each group, and after polishing with waterproof sandpaper up to grit #3000, they were immersed in 37 °C distilled water for 7 days. A microhardness tester (FM-300, Future-Tech, Kanagawa, Japan) was used to determine the Vickers hardness (HV). Four measurement points were selected at random for each sample, and a diamond indenter shaped as a regular square pyramid with a face angle of 136° was pressed against the surface of each specimen with a load of 1.96 N (0.2 kgf) for 10 s. The length d [mm] of the diagonal of the indentation at each measurement point was taken as the average of the two diagonals. The indenter pressing procedure and relative positioning of the indentations on the same specimen were performed in accordance with ISO 6507 [31], and the distances from the specimen edge to the center of each indentation, as well as the distances between indentations, were maintained at a minimum of three times the length of the diagonal. The HV value for each specimen was calculated using the following formula [31]:

$$Hv=0.1891\times \frac{F}{d^{2}}$$

where *F* is the applied load (unit: N), and *d* is the mean diagonal length of the indentation (mm).

### 2.3. Surface Characteristics and Surface Roughness of VPP Specimens at Each Build Angle

A white-light interference laser microscope was used to evaluate the surface topography of the unpolished CC and OT samples fabricated at various build angles. The specimens were designed in accordance with ISO 5139:2023 (Dentistry—Polymer-based composite machinable blanks) using the following dimensions: 14.0 mm in length, 4.0 mm in width, and 1.2 mm in thickness [32]. A total of 30 samples—five samples each of CC and OT specimens prior to polishing—were prepared under conditions of 0°, 45°, and 90° relative to the platform. These samples were observed from the top using the white-light interference laser microscope (VK-X3000, Keyence, Osaka, Japan), and 3D images were reconstructed. Furthermore, the surface roughness of the sample in any arbitrary area was measured without contact in a direction perpendicular to the long axis of the sample. Measurements were taken along multiple lines, with five lines per sample, for a total of 11 lines. The surface roughness parameters used were Ra (µm), Rz (µm), and RSm (µm).

### 2.4. Dimensional Accuracy of VPP Specimens at Each Build Angle

To evaluate the dimensional accuracy of the CC and OT specimens fabricated using various build angles, the width, thickness, and length of each specimen were measured before the polishing process. For each group, 20 samples (2.0 mm wide, 2.0 mm thick, and 25 mm long) were fabricated at build angles of 0°, 45°, and 90° relative to the platform. These specimens were designed in accordance with ISO 4049:2019 (Dentistry—Polymer-based restorative materials) [33]. A digital caliper was used to measure the dimensions of the samples fabricated using various materials and build angles. Measurements were taken near the center of the samples, avoiding the areas where supports were attached, and the differences between the measured values and respective set values were calculated. The differences in height (H), width (W), and length (L) were denoted as ΔH, ΔW, and ΔL, respectively.

### 2.5. Three-Point Bending Test

**Figure 1:**
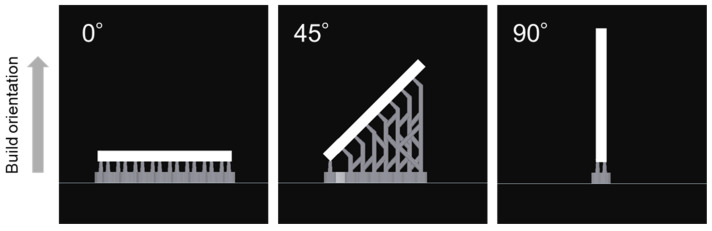
Design of vat photopolymerization samples fabricating at different build angles.

For each group, 15 test specimens measuring 2.0 mm in width, 2.0 mm in thickness, and 25 mm in length were prepared in accordance with ISO 4049:2019. For the CC and OT samples, the following three build angles relative to the platform were used: 0°, 45°, and 90° (Figure 1). After adjusting the dimensions for each molding angle, the corners of all the test specimens were rounded to avoid stress concentration, and they were finally polished to #3000 grit using waterproof sandpaper. The polished specimens were immersed in water at 37 °C for 7 days, and a three-point bending test was conducted in accordance with ISO 4049:2019. The distance between the supports was set to 20 mm. Using a universal testing machine (Auto graph AGS-J, Shimadzu, Kyoto, Japan), a load was applied to the center of the middle long side of the specimen at a crosshead speed of 1.0 mm/min, and the load value F [N] at which failure occurred was recorded. The three-point flexural strength σ_f_ (MPa) for each specimen was calculated using the following formula [33]:

$$\sigma_{f}=\frac{3FL}{2bh^{2}}$$

where *F* is the fracture load (N), *L* is the span length between supports (mm), *b* is the specimen width (mm), and *h* is the specimen thickness (mm).

In addition, using analysis software (Trapezium 2, Shimadzu, Kyoto, Japan), the flexural modulus (GPa) and fracture energy (N·mm) of each specimen during the three-point bending test were also evaluated.

### 2.6. Observation of the Fracture Surface After the Three-Point Bending Test

To investigate the fracture patterns of each group, the fracture surfaces were examined using a scanning electron microscope (SEM). After the three-point bending test, the fracture surfaces of the specimens were coated with osmium and examined using a field-emission SEM (S-4800, Hitachi, Tokyo, Japan) to evaluate the fracture characteristics of each material at different build angles. The secondary electron (low magnification) and backscattered electron (BSE) (high magnification) modes were used as observation modes.

### 2.7. Statistical Analysis

EZR version 1.61 (Saitama Medical Center, Jichi Medical University, Saitama, Japan) was used as the statistical analysis software. The normality and homogeneity of variance of each quantitative dataset (dimensional accuracy, surface roughness, mechanical parameters from the three-point bending test, and HV) were verified using the Shapiro–Wilk test and Bartlett’s test, respectively. Because several datasets did not fully satisfy the assumptions for parametric multifactorial analysis, a nonparametric approach was adopted to ensure robust comparisons among groups. The Kruskal–Wallis test was used to perform the statistical analysis and was followed by multiple comparisons between groups using the Bonferroni method. The significance level was set at 0.05.

A post hoc sample size justification was performed using G*Power version 3.1.9.7 (Heinrich-Heine-Universität Düsseldorf, Düsseldorf, Germany). Because G*Power does not directly support nonparametric tests such as the Kruskal–Wallis test, an approximate one-way ANOVA fixed-effects model was used as a conservative proxy (α = 0.05, power = 0.80, equal group allocation). Effect size estimates were derived from pilot measurements and variance observed in preliminary experiments. The calculated minimum sample size ranged from 2 to 4 specimens per group depending on the outcome variable. Therefore, the sample sizes used in the present study (n = 5–20 per group) were considered sufficient.

## 3. Results

### 3.1. Vickers Hardness

**Figure 2:**
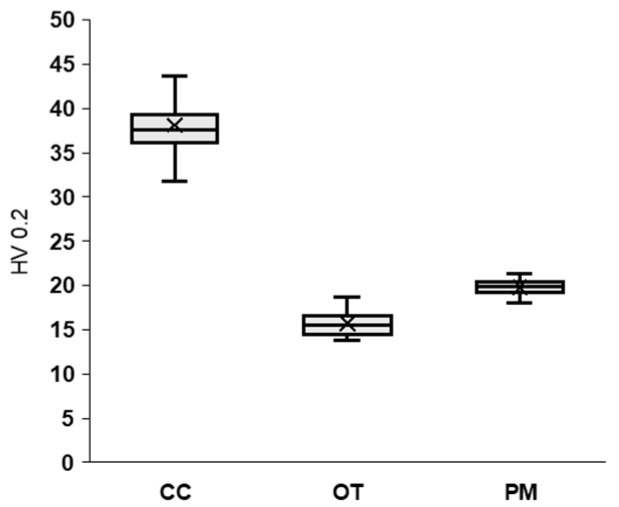
Vickers hardness of each material. Different letters indicate significant differences between groups (n = 20 per group, *p* < 0.05). CC: SprintRay Ceramic Crown, OT: SprintRay Onx Tough 2, PM: polymethyl methacrylate disc.

The mean HV values for each composite were as follows: CC, 38.1075 (95% confidence interval [CI]: 36.724–39.491); OT, 15.69915 (95% CI: 15.06–16.339); and PM, 19.8007 (95% CI: 19.407–20.194). Significant differences were observed between all groups. CC was nearly twice as hard as the other two materials, while the HV value of OT was lower than that of PM (Figure 2).

### 3.2. Surface Characteristics and Surface Roughness at Each Build Angle

**Figure 3:**
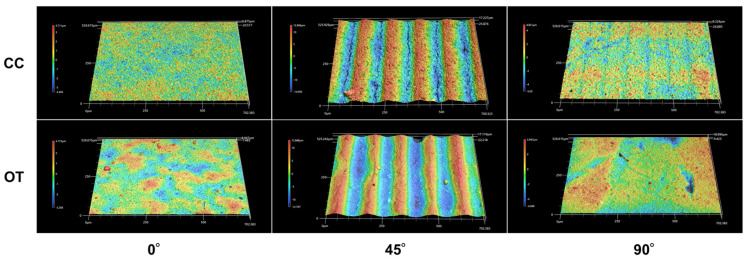
Representative surface topographies of CC and OT at each build angle. CC: SprintRay Ceramic Crown; OT: SprintRay Onx Tough 2.

**Figure 4:**
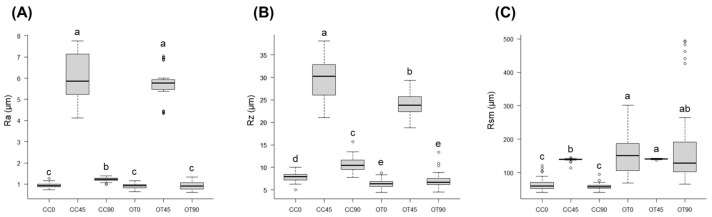
Surface roughness parameters of CC and OT at each build angle. (**A**) Arithmetic mean roughness (Ra); (**B**) Maximum height (Rz); (**C**) Mean element length (RSm). Different letters indicate significant differences between groups (n = 55 per group, *p* < 0.05). CC: SprintRay Ceramic Crown, OT: SprintRay Onx Tough 2.

Figure 3 shows the 3D surface topography patterns for CC and OT at various build angles (Figure 3). At a build angle of 0°, CC exhibited a relatively uniform surface topography, with no significant features observed in the distribution of surface irregularities. By contrast, OT exhibited a cluster-like distribution of protrusions and depressions, showing an island-like pattern of irregularities. At a 45° build angle, a distinct wave pattern with a period of approximately 140 µm appeared in all materials, resulting in alternating raised and recessed areas and a striped surface appearance. However, while CC exhibited a similar pattern of irregularities, its surface texture was generally rougher than that of OT. At a build angle of 90°, shallow, striped grooves spaced approximately 100 µm apart, matching the layer pitch, were observed on CC. By contrast, OT did not exhibit this striped pattern; instead, protrusions and depressions were distributed irregularly. On comparing the two materials, CC generally exhibited a rough surface structure characterized by fine irregularities, whereas OT tended to exhibit a surface morphology featuring distinct protrusions and depressions, accompanied by undulations. Figure 4 shows the surface roughness parameters for CC and OT at various build angles. Regarding the Ra and Rz values, which indicate the vertical component, the 45° build angle resulted in significantly higher values for both materials, reflecting the striped irregularities caused by the wave pattern. On the other hand, while the Rsm values indicating the horizontal component converged to approximately 140 µm for all materials at a build angle of 45°, the Rsm values at the build angles of 0° and 90° reflected the influence of surface undulations in the OT data recorded at these specific angles, resulting in the values of OT being greater than those of CC; additionally, a tendency toward greater variability was observed in the OT data.

### 3.3. Dimensional Accuracy at Each Build Angle

**Figure 5:**
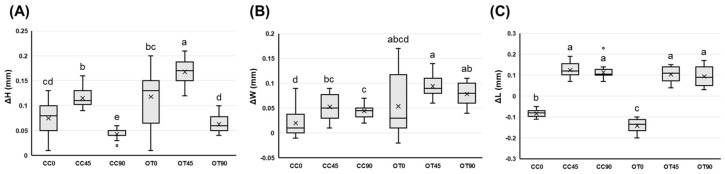
Dimensional accuracy of test specimens in accordance with ISO 4049. (**A**) Average difference between measured and specified values in the height direction (H); (**B**) Average difference between measured and specified values in the width direction (W); (**C**) Average difference between measured and specified values in the length direction (L). Different letters indicate significant differences between groups (n = 20 per group, *p* < 0.05).

For each material, ΔH and ΔW tended to be larger at a build angle of 45°, suggesting a greater tendency for overbuilding in both the height and width. This trend was even more pronounced in the case of ΔH. Furthermore, when comparing ΔH and ΔW across the materials at the same build angle, a trend of OT values being greater than CC values was observed, suggesting that OT may yield slightly lower dimensional accuracy. Regarding ΔL, while the 0° build angle resulted in a value lower than the set value for all materials, the 45° and 90° build angles resulted in higher values than the set value; no difference was observed in ΔL between CC and OT at the same build angle. Based on the above results, this VPP system tends to overbuild in the z-axis direction relative to the platform regardless of the type of material used, and the build angle may affect dimensional accuracy in each direction (Figure 5).

### 3.4. Mechanical Properties of Each Group During the Three-Point Bending Test

**Figure 6:**
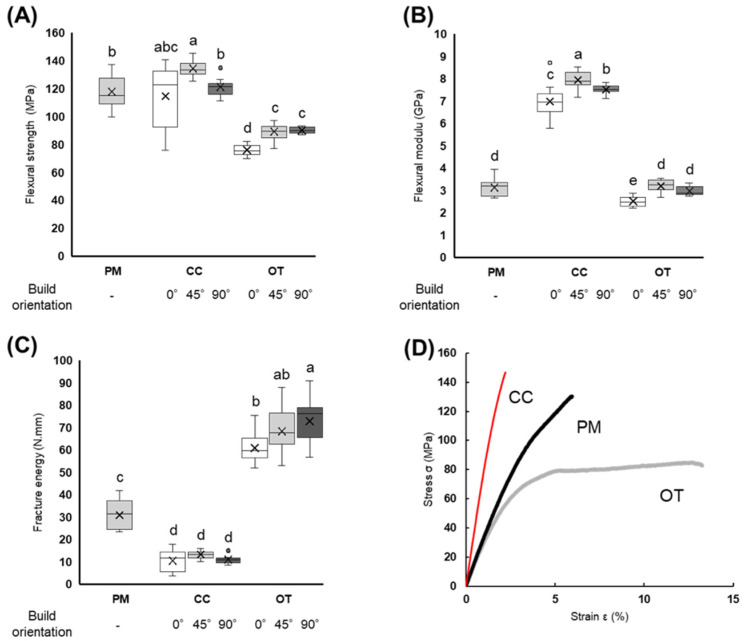
Mechanical parameters of each group in the three-point bending test. (**A**) Flexural strength; (**B**) Flexural modulus; (**C**) Energy to fracture; (**D**) Representative stress–strain curves at a 45° build angle. Different letters indicate significant differences between groups (n = 15 per group, *p* < 0.05). CC: SprintRay Ceramic Crown, OT: SprintRay Onx Tough 2, PM: PMMA disc.

Figure 6A–C show the mechanical parameters obtained using the three-point bending tests for each material at different build angles. For flexural strength, the values were as follows: PM, 117.944 ± 11.2024 MPa; CC0, 114.844 ± 23.5484 MPa; CC45, 134.656 ± 6.02466 MPa; CC90, 121.344 ± 7.21654 MPa; OT0, 76.1875 ± 4.03119 MPa; OT45, 89.3063 ± 5.3343 MPa; and OT90, 90.2438 ± 2.08964 MPa. Across all the materials, the relationship was generally PM ≈ CC > OT. In the analysis by build angle, CC tended to exhibit slightly higher flexural strength at a 45° build angle compared to at the other angles, while OT tended to exhibit slightly higher flexural strength at 45° and 90° compared to at 0° (Figure 6A). The values for flexural modulus were as follows: PM, 3.13273 ± 0.37544 GPa; CC0, 6.99314 ± 0.671127 GPa; CC45, 7.94126 ± 0.392496 GPa; CC90, 7.52206 ± 0.183584 GPa; OT0, 2.51264 ± 0.222279 GPa; OT45, 3.20136 ± 0.256753 GPa; and OT90, 2.96788 ± 0.20008 GPa. Across all materials, the relationship was generally CC > PM ≒ OT, and when analyzed by build angle, a trend of 45° > 90° > 0° was observed for both CC and OT (Figure 6B). The values for fracture energy were as follows: PM, 30.9263 ± 6.43972 N·mm; CC0, 10.53 ± 4.59 N·mm; CC45, 13.32 ± 1.73 N·mm; CC90, 11.05 ± 1.98 N·mm; OT0, 60.93 ± 6.28 N·mm; OT45, 68.41 ± 9.57 N·mm; and OT90, 72.85 ± 9.57 N·mm. Among the materials, the order of properties was OT > PM > CC; CC exhibited low energy to fracture and brittle behavior, whereas OT exhibited high resistance to fracture. When analyzed by build angle, CC showed a trend of 45° > 90° > 0°, while OT showed a trend of 90° > 45° > 0° (Figure 6C). Under the conditions of this experiment, the results suggest that, in general, a build angle of 45° is the most acceptable condition on considering all mechanical parameters. Furthermore, to provide a visual understanding of the mechanical properties of each material, stress–strain curves were plotted for the samples made from the various materials. As a representative example, Figure 6 shows the stress–strain curves for the various materials when the VPP resin specimens were fabricated at a 45° angle. While CC exhibited a relatively high flexural modulus and behaved similarly to a brittle material, OT yielded under relatively low stress accompanied by plastic deformation and subsequently exhibited viscous behavior until fracture. OT required a significant amount of energy to reach the point of fracture. Furthermore, although PM exhibited initial deformation similar to that exhibited by OT, its elastic deformation phase was relatively long, resulting in mechanical behavior values intermediate between those of CC and OT (Figure 6D).

### 3.5. Observation of the Fracture Surfaces of Each Specimen After the Three-Point Bending Test

**Figure 7:**
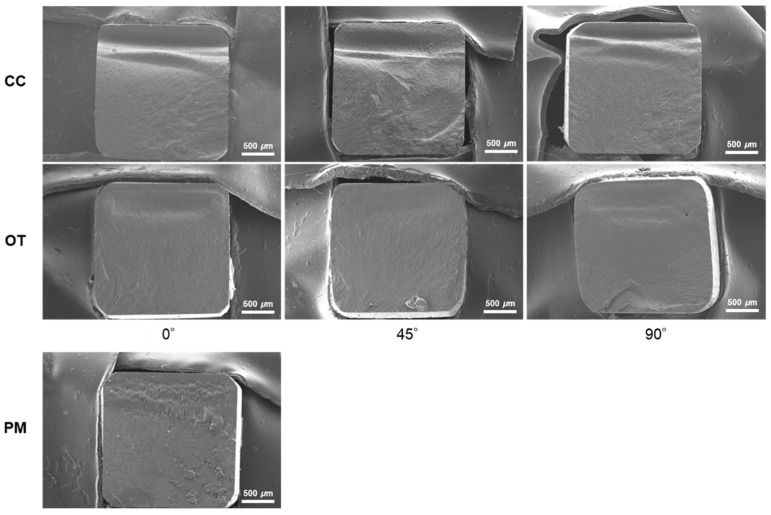
Representative scanning electron microscope images of the fracture surfaces of each specimen after bending tests, categorized by build angle (×35). CC: SprintRay Ceramic Crown, OT: SprintRay Onx Tough 2, PM: PMMA disc.

**Figure 8:**
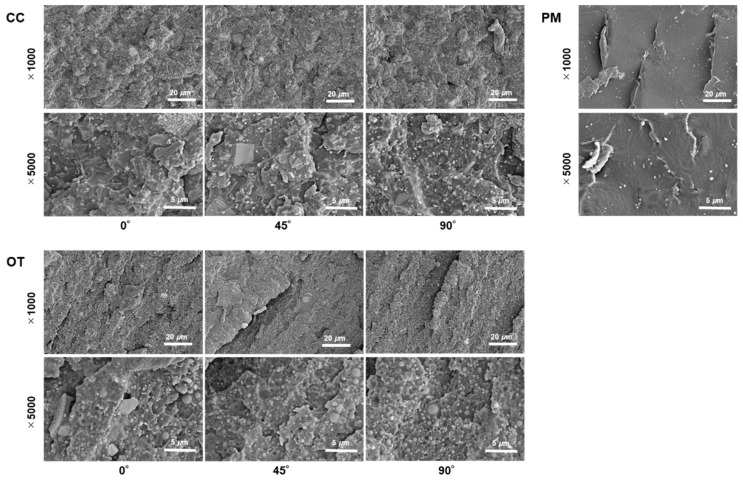
High-magnification scanning electron microscope images of the vicinity of the fracture initiation point in each specimen after bending tests, categorized by build angle (×1000, ×5000). CC: SprintRay Ceramic Crown, OT: SprintRay Onx Tough 2, PM: PMMA disc.

Figure 7 shows the macroscopic fracture surface after a typical three-point bending test, and Figure 8 shows the microscopic fracture surface near the fracture initiation point. In the macro fracture analysis (×35), cracks in the CC specimen propagated from the region of tensile stress concentration (lower part of the specimen) as the starting point, regardless of the build angle. Compressive stress developed on the load-bearing side (upper part of the specimen), leading to the final fracture, and a distinct step was observed in that region. Furthermore, when examined by build angle, while samples exhibited relatively smooth fracture surfaces at 0° and 90°, a 3D complex fracture surface morphology was observed at 45°, suggesting that the direction of crack propagation was dispersed. Conversely, OT exhibited a similar pattern at all build angles and showed a uniformly homogeneous and smooth fracture surface. In addition, the step at the point of compressive stress concentration (top of the specimen) was less distinct than that in CC. On observing the direction of crack propagation by build angle, OT0° and OT45° exhibited a pattern of vertical propagation, whereas OT90° showed a tendency for the crack to propagate horizontally in some areas. PM exhibited ductile fracture characteristics typical of general-purpose resins, and macroscopic fracture surface observation revealed irregularities caused by overall elongation (Figure 7).

In the micro-fracture analysis (×1000, ×5000), a composite of fine, amorphous nanofillers and a resin matrix were observed in the CC specimens; moreover, since the fracture surface exhibited a dimple-like pattern under high magnification, the resin portion suggested a ductile fracture pattern. However, it was predicted that cracks would propagate due to a chain of internal defects at any build angle, suggesting the possibility that the structure could exhibit brittle behavior. By contrast, in the low-magnification image (×1000), the direction of fracture propagation was clearly visible at all build angles, suggesting that this finding reflects the viscoelastic properties of the matrix resin composition. Furthermore, at high magnification (×5000), the filler morphology differed significantly from that of CC, as submicron-sized spherical fillers were observed along with amorphous nanofillers. Moreover, at the fracture surface, delamination was observed at the interface between the spherical filler and matrix resin. In the post-mortem examination, the microstructure near the fracture initiation point revealed a smooth fracture surface with a structure resembling a river pattern, exhibiting characteristics similar to those of brittle fracture. In addition, the filler component showed only scattered, minute amorphous particles that appeared to be pigments; no clear evidence was found indicating that these could serve as stress initiation points. Furthermore, virtually no internal defects were observed; only discontinuous cracks were noted. These findings were considered to confirm the homogeneity of this material as a structural component (Figure 8).

## 4. Discussion

Among the VPP resins used in this study, CC exhibited superior hardness and elastic modulus values compared to the control material, PM, but tended to have lower energy to fracture. Meanwhile, OT tended to exhibit slightly lower dimensional accuracy and stability after underwater storage; furthermore, although it showed superior energy to fracture compared to the other two materials (CC and OT), its hardness and flexural strength were lower than those of the reference material, PM. Furthermore, laser microscopic observations of the sample surface prior to polishing revealed distinct surface texture patterns at each build angle, with CC exhibiting a relatively uniform and rough surface texture. Conversely, protrusions and depressions appeared in clusters on the OT surface, resulting in a tendency for the surface to exhibit undulations. Furthermore, the differences in surface texture patterns between these materials and across different build angles were found to correspond to the surface roughness parameter values in each group. Dimensional accuracy varied depending on the build angle, with each dimension showing different trends; generally, there was a tendency toward overbuilding in the direction perpendicular to the platform. Furthermore, based on the mechanical parameters obtained from the bending tests at different build angles, the results suggest that, from the perspective of mechanical performance, a build angle of 45° may represent a favorable condition for both CC and OT materials. However, although the 45° build angle tended to provide favorable mechanical properties, it also showed increased surface roughness and dimensional deviation. Therefore, the optimal build angle should be selected according to the intended clinical application, considering the balance between mechanical strength, dimensional accuracy, and surface quality. Based on the above findings, the null hypothesis of this study was rejected.

First, as a representative mechanical property, the HV value of each material was measured. The hardness of a material is generally known to correlate with its wear resistance [34,35]. Therefore, in clinical applications in which medium- to long-term follow-up is required for provisional restorations, hardness is considered an important parameter from the perspective of maintaining the occlusal vertical dimension and mandibular position [36,37]. The results of this study show that hardness increased in the order of CC > PM > OT, with CC exhibiting a significantly higher HV value than the other two materials. CC consists of a filler content of approximately 50 wt% and matrix resin component primarily composed of multifunctional monomers [38]; it is believed to exhibit a higher HV value than the PM group, which uses PMMA as the main raw material. Previous reports have indicated that the hardness of general purpose composite resins ranges from approximately 14.4 to 57.2 HV [39]; accordingly, the hardness of CC was generally comparable to these values. By contrast, although OT is a composite resin material containing filler, it exhibited lower hardness than PM, with its hardness falling near the lower limit of previously reported values [39]. Based on the above findings, it is clear that the mechanical properties of the two types of VPP resins (CC and OT) used in this experiment are fundamentally different. Furthermore, since the resin composition of OT differs significantly from that of CC [40], it is possible that these differences in material composition contributed to the mechanical properties of the fabricated objects.

In addition, the build angle in AM technology could potentially affect various parameters of the fabricated objects. Therefore, in this study, in addition to comparing the physical properties of each material, the surface profile and roughness parameters, dimensional accuracy, and mechanical properties during bending tests were evaluated for the VPP resin materials at build angles of 0°, 45°, and 90°.

Surface characteristics and surface roughness in crown restoration materials may be associated not only with poor tactile sensation but also with restoration discoloration and plaque retention [29,40]. Furthermore, it has been suggested that in hard materials, such as zirconia, inadequate surface finish resulting from poor polishing affects wear on the opposing dentition; thus, an appropriate surface finish is crucial not only for the prognosis of the prosthesis but also for the long-term management of oral function [41]. Wang et al. evaluated various VPP-based composite resin materials by examining surface roughness, cytotoxicity, and bacterial adhesion for each system [29]. Nam et al. also suggest that in various VPP resins, controlling surface roughness through surface treatments, such as glazing and coating, reduce protein adsorption and contribute to the resistance against staining [40]. Furthermore, the build angle in VPP resin may be related to surface roughness; Shim et al. investigated the accuracy and mechanical properties of VPP resin at various build angles, as well as various surface characteristics—including surface roughness and fungal adhesion [20]. In this study, build angles of 0°, 45° and 90° were used. At a build angle of 45°, alternating raised and recessed areas appeared in a wave pattern with spacing of approximately 140 µm in both CC and OT materials. This is thought to correspond to the diagonal ratio (1:√2) when the layer pitch is set to 100 µm, resulting in the appearance of a periodic pattern on the surface; this finding is consistent with the observation that the Rsm values at a 45° build angle converged within a certain range for both materials. Conversely, surface roughness measurements across the direction of this waveform pattern revealed that the Ra and Rz parameters, which represent vertical roughness, were significantly higher at a build angle of 45°. Furthermore, on observing the surface morphology of each material at build angles of 0° and 90°, CC exhibited a rough and homogeneous surface, whereas OT showed an irregular, island-like pattern of surface irregularities. Additionally, consistent with the above findings, the Rsm values for OT at 0° and 90° were higher than those for CC and exhibited an irregular range. These findings are thought to reflect the surface irregularities of the OT sample caused by an irregular, island-like pattern of protrusions and depressions. The shape and dispersion of the filler, or the accumulation of filler on the sample surface, may be factors that contribute to this phenomenon. As noted above, this experiment showed that surface characteristics and surface roughness parameters tended to vary depending on the material or build angle. However, when considering the fabrication of prostheses, since areas with varying angles of inclination coexist within the same device, it is considered essential to perform comprehensive polishing, coating, and other appropriate surface finishing processes in any case.

Inadequate dimensional accuracy and mechanical strength can lead to poor fit or the need to refabricate prostheses. Previous studies have reported that the build angle affects mechanical strength and dimensional accuracy in VPP systems [20,21,22,23]. Shim et al. have shown that while a build angle perpendicular to the platform (90°) tends to yield superior dimensional accuracy, it may result in reduced mechanical strength [20]. Furthermore, Tahayeri et al. have pointed out that while dimensional accuracy in the width and length directions was excellent at a build angle of 90°, the error ratio in the thickness direction was high [42]. This study demonstrated a tendency toward significant overbuilding in the thickness direction at build angles of 0° or 45°, while showing relatively superior dimensional accuracy at 90°, consistent with the findings reported by Shim et al. Previous reports have indicated that when the build angle is set to 0° in the SprintRay system, filler deposition is observed on the support contact surface in the thickness direction, suggesting a possible association with overbuild [30,43]. On the other hand, while the length dimension (ΔL) tended to be overbuilt at 45° and 90°, the 0° dimension was actually shorter than expected. Therefore, the difference in the directionality of the dimensional accuracy of ΔL between the build angles of 0° and 45°—which are unrelated to the support attachment surface—cannot be easily explained by the aggregation of resin slurry or filler at the support attachment site. Within the scope of this experiment, it was demonstrated that overbuilding may occur at build angles where the platform has a z-axis component. However, the dimensional accuracy data in this experiment were obtained solely by measuring two-dimensional distances near the center of the sample and should be interpreted within the scope of these limitations. In future research, it will be necessary to scan the entire sample and overlay it with the design data to evaluate the overall 3D accuracy of the sample at each build angle.

In this study, the mechanical parameters of the PMMA disc material (PM) and VPP resin materials (CC and OT) at various build angles were compared during bending tests. In general, it is known that adding inorganic fillers to resin materials improves their mechanical properties [26]. Furthermore, the build angle used in VPP resins may affect mechanical strength depending on the relationship between the load direction and layer orientation [44]. In this study, a build angle of 45° yielded relatively high values of both three-point flexural strength and elastic modulus in CC and OT. Sahin et al. reported that a molding angle of 45° yielded the highest flexural strength in resin used for occlusal splints [45]. The results of this study showed trends in mechanical properties that were generally similar to those reported by Sahin et al. Furthermore, when evaluated against the flexural strength criteria of ISO 4049:2019, CC met the criteria for Type 1, Class 2, and Group 1 (≥100 MPa), whereas OT fell below 100 MPa, falling within the threshold (≥80 MPa) for other classifications under Type 1. On the other hand, when PM was used as the reference, CC exhibited superior hardness and elastic modulus and showed brittle properties that made it resistant to deformation under stress, whereas OT, despite containing fillers, showed hardness and elastic modulus values equivalent to or lower than those of PM. Meanwhile, OT demonstrated significant fracture energy and was shown have excellent fracture resistance. These differences in mechanical properties were predicted to reflect the overall properties of the composite structure, depending not only on the matrix resin composition and presence or absence of fillers, but also on factors, such as the number of internal defects that could serve as stress initiation points due to the processing method, as well as the shape and distribution of the fillers.

**Figure 9:**
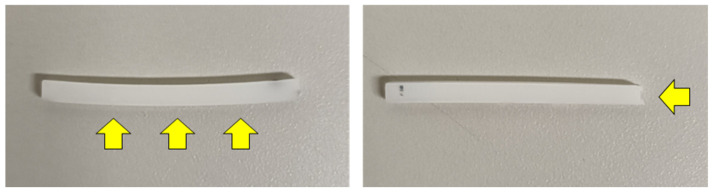
A typical OT sample after 7 days of immersion in water. The yellow arrows indicate the support-contacting surfaces. The OT samples tended to deform in the direction of the support-contacting surfaces. OT: SprintRay OnX Tough 2.

Based on observations of the fracture surfaces after the bending test, the CC specimen fabricated at a 45° build angle exhibited a fracture surface with more irregularities than those on specimens fabricated at other build angles (Figure 7). This may have delayed the failure behavior because the layers formed by the 3D printing process were oriented at an angle to the direction of the load, resulting in complex stress distribution. Furthermore, under high magnification, as a structural body, the CC specimen appeared to exhibit behavior closely resembling that of brittle fracture likely because of a chain of internal voids, uneven filler distribution, or delamination between the matrix resin and filler (Figure 8). By contrast, OT exhibited a filler morphology that was completely different from that of CC, with spherical fillers observed in addition to amorphous silica. These fillers are thought to be added to adjust the resin’s physical properties, such as formability; due to the spherical shape of these fillers, they are less likely to act as stress initiation points, which may have contributed to the high energy to fracture. On the other hand, the difference in filler particle size between CC and OT may have affected the surface profile and dimensional accuracy of the cured product as a result of the viscosity of the resin slurry and density distribution of the filler (Figure 8). However, the present SEM observations were primarily qualitative, and no additional quantitative microstructural or chemical analyses were performed. Therefore, the proposed mechanisms of crack propagation and filler-related behavior should be interpreted with caution. Furthermore, in the OT samples, although these findings are purely qualitative, there was a tendency for greater deformation after storage in water. The OT0° and 45° models deformed into a convex shape relative to the support surface, while the 90° model exhibited a different deformation pattern (Figure 9). Although qualitative deformation tendencies were observed in OT after water storage, quantitative evaluation of dimensional changes and aging-associated degradation was beyond the scope of this study. Consequently, only at a build angle of 90°, a difference in the crack propagation direction was observed in the macro fractography analysis (Figure 8), which may explain the tendency for higher fracture energy at 90° in the analysis by build angle (Figure 6C). Moreover, these characteristics may be associated with differences in resin composition. According to the manufacturer’s material information, OT contains hydrophilic HEMA as a matrix component, which may contribute to greater water sorption compared with CC. However, because chemical composition analyses were not directly performed in the present study, this interpretation remains speculative. Further investigations using compositional analyses such as FTIR or EDS analysis are required to clarify the mechanisms underlying these material-dependent behaviors. Furthermore, differences in filler morphology and distribution observed in SEM images may also be related to the distinct elastic modulus and stress–strain behaviors of the materials. Therefore, while OT exhibited high fracture resistance, it may also be more prone to plastic deformation, and material selection should be based on comprehensive evaluation of these mechanical characteristics. In addition, the final properties of photopolymerized materials may be influenced by post-curing conditions, including light intensity, curing duration, and thermal environment. In the present study, post-curing was standardized according to the manufacturer-recommended protocol to minimize procedural variability; however, the specific influence of post-curing parameters was beyond the scope of this study.

In this study, we selected VPP-processed hard resin materials for dental crowns—materials that are expected to be applied in the fabrication of fixed dental prostheses and have been approved as managed medical devices. Subsequently, physical properties were compared with those of existing materials, and the performance of each material was evaluated based on the build angle. From a clinical perspective, the relatively high hardness and flexural strength of CC may be advantageous for maintaining occlusal morphology and wear resistance in definitive single-unit restorations. However, its lower fracture energy suggests a greater susceptibility to brittle failure under excessive loading. In contrast, OT demonstrated high fracture resistance but lower hardness and flexural strength, which may limit its suitability for definitive restorations subjected to high occlusal loads, while making it potentially useful for provisional restorations or applications requiring greater resistance to catastrophic fracture. Therefore, material selection should be based on the anticipated functional demands and intended duration of use.

Currently, in Japan, treatment using complete dentures fabricated using a photopolymerization-based 3D printing system for denture bases is covered by health insurance [46]. Given this trend, it is also considered urgent to accumulate evidence for the application in the fabrication of fixed dental prostheses. The results of this study provide valuable insights into the VPP crown-grade composite resin materials currently available in Japan. However, this study has certain limitations. First, we were unable to perform a 3D evaluation of dimensional accuracy or to compare it with that in CAD-CAM milling methods. The present dimensional analysis was limited to linear measurements obtained using digital calipers and may not fully reflect three-dimensional deviations across the entire specimen geometry. In future studies, full-field three-dimensional evaluation using optical scanning and STL superimposition analyses may provide a more comprehensive assessment of trueness and precision. Furthermore, stress behavior was not verified during function in a form resembling an actual prosthesis, nor was a verification of material degradation over time or dimensional accuracy conducted. In clinical restorations, stress distribution may be substantially influenced by restoration geometry, connector dimensions, occlusal morphology, and localized stress concentration areas. Therefore, the mechanical behavior observed in standardized bar-shaped specimens may not directly reflect the performance of actual crowns or fixed dental prostheses under complex intraoral loading conditions. Brittle materials with high stiffness may exhibit different fracture behavior in restorations containing sharp internal contours or thin sections. Accordingly, the present findings should be interpreted as fundamental material data rather than direct predictors of long-term clinical performance. In addition, the present study evaluated the materials only under static loading conditions, and cyclic mechanical fatigue simulating repeated masticatory loading was not performed. Thermal aging protocols, such as thermocycling, as well as long-term water storage were also not included; therefore, the long-term durability and degradation behavior of these materials under oral conditions remain unclear. Thus, additional investigations incorporating long-term water storage [47], thermocycling [48], and fatigue loading [49] are necessary to better predict long-term clinical performance. For AM-based dentistry to be integrated as a core component of dental treatment rather than merely serving as an alternative to conventional methods, further research is needed, specifically involving improvements and evaluations of materials and systems, the accumulation of clinical data, and verification of the scope of application.

## 5. Conclusions

Within the scope of this study that tested the two types of VPP resins approved as medical devices in Japan, CC exhibited superior hardness and elastic modulus compared with the control material, PMMA discs (PM), but had lower energy to fracture. By contrast, OT tended to exhibit slightly lower dimensional accuracy and stability after underwater storage, but it demonstrated superior energy to fracture compared to the CC and PM, showing high resistance to fracture. However, its hardness and flexural strength were lower than those of the control material, PM, and it underwent plastic deformation easily under external force. Furthermore, surface properties, surface roughness, dimensional accuracy by build angle, and mechanical parameters tended to vary depending on the material and build angle. Based on the above findings, when considering the clinical application of the VPP resin materials used in this experiment, careful consideration must be given to material selection and intended use in each specific case, taking into account the surface characteristics, dimensional accuracy, and mechanical properties of each material at different build angles.

## Figures and Tables

**Table 1:** Materials used in this study.

Material (Code)	Manufacturer	Type of Material	Composition	Lot. No.
Ceramic Crown (CC)	Sprintray, CA, USA	VPP resin	methacrylate monomers, oligomers, acrylic monomers, photo initiator, inorganic fillers, etc.	S24G033
OnX Tough 2 (OT)	Sprintray, CA, USA	VPP resin	urethane dimethacrylate, amorphous silica, urethane methacrylate, hydroxyethyl methacrylate, ytterbium fluoride, trimethylbenzoyl diphenylphosphine oxide, silica dimethyl silylate, etc.	S2513020
PMMA disc (PM)	Yamahachi, Aichi, Japan	CAD-CAM resin disc	polymethylmethacrylate, carbon black, ferric oxide, titanium dioxide, etc.	UE02

VPP: vat photopolymerization, PMMA: polymethyl methacrylate, CAD-CAM: computer-aided design and computer-aided manufacturing.

## Data Availability

The original contributions presented in this study are included in the article. Further inquiries can be directed to the corresponding author.

## Abbreviations

The following abbreviations are used in this manuscript:

AM	additive manufacturing
VPP	vat photopolymerization
CC	SprintRay Ceramic Crown
OT	OnX Tough 2
PMMA	polymethyl methacrylate
STL	standard tessellation language
HV	Vickers hardness
CI	confidence interval
SEM	scanning electron microscopy
BSE	backscattered electron
Ra	arithmetic mean roughness
Rz	maximum height roughness
RSm	mean element length
ΔH	deviation in specimen height from the designed value
ΔW	deviation in specimen width from the designed value
ΔL	deviation in specimen length from the designed value
